# Estimation of 3D Knee Joint Angles during Cycling Using Inertial Sensors: Accuracy of a Novel Sensor-to-Segment Calibration Procedure Based on Pedaling Motion

**DOI:** 10.3390/s19112474

**Published:** 2019-05-30

**Authors:** Sébastien Cordillet, Nicolas Bideau, Benoit Bideau, Guillaume Nicolas

**Affiliations:** 1 M2S Laboratory (Movement, Sports & Health), University Rennes 2, ENS Rennes, 35170 Bruz, France; sebastien.cordillet@univ-rennes2.fr (S.C.); nicolas.bideau@univ-rennes2.fr (N.B.); benoit.bideau@univ-rennes2.fr (B.B.); 2 MIMETIC–Analysis-Synthesis Approach for Virtual Human Simulation, INRIA Rennes–Bretagne Atlantique, IRISA_D6–MEDIA ET INTERACTIONS, 35000 Rennes, France

**Keywords:** inertial sensors, misalignment correction, accuracy, pedaling motion, sensor-to-segment calibration, 3D knee joint angles

## Abstract

This paper presents a novel sensor-to-segment calibration procedure for inertial sensor-based knee joint kinematics analysis during cycling. This procedure was designed to be feasible in-field, autonomously, and without any external operator or device. It combines a static standing up posture and a pedaling task. The main goal of this study was to assess the accuracy of the new sensor-to-segment calibration method (denoted as the ‘cycling’ method) by calculating errors in terms of body-segment orientations and 3D knee joint angles using inertial measurement unit (IMU)-based and optoelectronic-based motion capture. To do so, 14 participants were evaluated during pedaling motion at a workload of 100 W, which enabled comparisons of the cycling method with conventional calibration methods commonly employed in gait analysis. The accuracy of the cycling method was comparable to that of other methods concerning the knee flexion/extension angle, and did not exceed 3.8°. However, the cycling method presented the smallest errors for knee internal/external rotation (6.65 ± 1.94°) and abduction/adduction (5.92 ± 2.85°). This study demonstrated that a calibration method based on the completion of a pedaling task combined with a standing posture significantly improved the accuracy of 3D knee joint angle measurement when applied to cycling analysis.

## 1. Introduction

The quantification of 3D kinematical parameters such as body segment orientations and joint angles is important in the monitoring of cycling to provide relevant biomechanical parameters associated with performance optimization and/or injury prevention [1,2]. For instance, some studies have observed that increases in maximum knee flexion (from 25° to 35°) could cause a reduction in cycling economy and anaerobic mean power output [3], which are commonly known to affect performance. The importance of the knee joint to performance can also be illustrated by an increasing contribution of knee moment to performance as intensity increases [1]. Simultaneously, the knee is considered to be one of the most common sites of injuries in cyclists, especially for overuse injuries [4]. Traumatic risk can be exacerbated by multiple factors, such as non-sagittal knee deviations (e.g., knee abduction/adduction or internal external rotations) [1,2,5], excessive knee flexion angles resulting from a low saddle height, or excessive knee extension from a higher saddle height [6,7,8]. Therefore, knee joint kinematics is a subject of special attention in cycling biomechanics. Numerous experiments based on optoelectronic motion capture have been conducted in the laboratory to analyze kinematical variables such as knee rotations during cycling [9]. However, the assessment of kinematics in real conditions during training or competition is a challenging task, especially since conventional optoelectronic motion capture systems suffer from major drawbacks in this regard. Indeed, performing 3D motion capture outside of the laboratory requires a large number of pedaling cycles and large capture volumes, which makes it challenging to use conventional optical motion capture systems [10]. Therefore, this analysis was often limited due to the restricted field of view associated with the number of cameras. Moreover, the acquisition to reconstruction process associated with marker-based optical systems may be cumbersome and time consuming, thereby reducing its effectiveness as a feedback tool and limiting its application in practice. Finally, reliable 3D quantitative camera-based analysis can only be performed off-line in a restricted area, and thus, cannot be used by coaches during training sessions for detecting and immediately correcting technical mistakes.

To overcome these limitations, recent advances in the development of microelectromechanical systems (MEMS) through wearable measurement systems, such as inertial measurement units (IMU), seem to be a relevant solution for in situ cycling analysis as they allow a continuous data acquisition process throughout a cycling exercise. As IMUs are small, lightweight, and do not interfere with the execution of movements during measurements, they are now increasingly used for sports and clinical biomechanics [11,12,13]. An inertial measurement unit embeds a 3D accelerometer and a 3D gyroscope, which are eventually linked to a magnetometer. The accelerometer provides the linear accelerations of the sensor, the gyroscope gives the angular rates, while the magnetometer measures the magnetic field, making it a source of information concerning the orientation of the IMU as regards to the magnetic north. Generally, combining these three types of information (accelerometer, gyroscope, magnetometer) through sensor-fusion algorithms [14,15] is proving to be a powerful method to obtain the 3D orientation of the IMU sensor in the absence of ferromagnetic disturbances in the close environment or under homogeneous earth magnetic field conditions [16]. Concerning cycling, Cockroft [17] demonstrated that IMUs may experience significant magnetic interferences of up to a 50% deviation in intensity near the pedals and handlebars, causing significant errors in kinematic measurements. In such a case, an alternative approach would involve combining the magnetometer and inertial sensors and correcting the magnetic field disturbances by developing specific sensor fusion algorithms [15]. However, the estimates of the magnetic north from two IMUs may differ due to discrepancies in the magnetometer data [18,19], which can be a major limitation of using a magnetometer despite ad hoc corrective methods. Another approach completely omits the magnetometer data to assess IMU orientation using only a 6-DOF measurement (3D linear accelerations and 3D angular rates), although it generally requires dedicated methods for biomechanical relevance [20,21,22,23].

Beyond the common problem of the drift related to the integration of gyroscope data, one of the major issues in joint kinematics assessment using IMU devices lies is the misalignment of sensor axes with the anatomical body segment axis, which is not straightforward [12]. This topic is crucial when attempting to provide functionally meaningful 3D joint kinematics based on inertial sensors. Usually, this problem is solved by performing calibration procedures for the calculation of the relative orientation between IMU frames and body segment frames. In other words, body segment orientation can be assessed from the IMU orientation and relative orientation of the body segment frame with respect to the sensor frame. This relationship is assumed to be time-invariant and, hence, can be accomplished once the sensors are mounted on the segment using ad hoc calibration procedures. Thus, several approaches have been developed to determine the sensor frame’s orientation with respect to the body segment frame [12]. The simplest way to align the sensor frame with the segment is to perform it manually [24], with the accuracy of this alignment being directly dependent on the ability of the operator to fix the sensor. Thus, Picerno [25] used a calibration device to pinpoint anatomical landmarks for identification of the segment axes. However, due to the requirement of an external experienced operator with anatomic and palpation capacities, such an approach compromises its use for the purposes of an easy self-calibration in a daily training routine. Other calibration procedures rely on functional methods that involve predefined movements and/or static postures of the user to define some joint axes of rotation or the body segment axes that are known during the specific tasks. Based on more arbitrary movements, alternative methods using kinematic models and constraints allow the identification of the joint axis coordinates [23,26,27,28]. Whereas these latter methods consider non-linear optimization algorithms, a recent computationally-efficient method has been proposed to produce estimates of the axis of rotation through principal component analysis [29]. 

Using an IMU including a magnetometer, the simplest approach consists of holding a standardized posture, such as an N-pose or T-pose, during which all body segment axes are calibrated with the same posture [30]. In the case where the magnetometer data are corrupted, functional methods using a combination of postures may be employed based on the accelerometer and gyroscope only. To date, these calibration methods have been conducted either on purely static postures, purely dynamic movements, or by combining both static and dynamic tasks. Calibration methods based on static postures are based on an accelerometer, which is used to obtain the orientation of the gravity vector when no acceleration of the IMU frame exists, that in turn is used as an estimator of the body segment axis during specific static postures [14,31,32,33]. Calibration methods based on dynamic movements use gyroscopic data that allow the calculation of the body segment axes when performing specific motions to isolate the functional rotation axis [33,34,35,36]. In such a context, dynamic tasks assume that motion occurs strictly in an anatomical plane, and thus, the rotation axis is defined as being directly orthogonal to this plane [37]. The functional method proposed by Palermo [31] combined two postures: a standing posture (similar to the N-pose) and a lying down posture. Other functional methods use dynamic motions to estimate the body segment axes. For example, Favre et al. proposed a calibration for the shank, based on a flexion/extension movement of the knee and a rotation of the shank in a frontal plane [34], which enabled an assessment of the medio-lateral and anteroposterior axes, respectively. Finally, two studies [32,37] proposed combining static and dynamic tasks to perform the calibration. Both studies used the standing up posture to define the longitudinal axis of the body segment in the sensor frame. 

It has been demonstrated that the accuracy of body segment orientation, as well as joint angle measurement, are impacted by the choice of calibration tasks [30,33,38]. Indeed, the validity of such procedures is based on the estimation of the accuracy, which has not been thoroughly tested with regard to lower limb motions, and, more specifically, in cycling. However, misalignment between the segment frame and the sensor frame is crucial and it affects the accuracy of joint angle estimation. Indeed, Brennan et al., [39] showed a direct relationship between perturbation of the body segment orientation and joint angle measurement accuracy. Previous studies showed that accuracy of the body segment orientation is dependent on the calibration procedures that can be achieved either in an autonomous or assisted way, bearing in mind that the ability to perform self-calibration procedures may be important for easy use in-field. Therefore, Robert-Lachaine et al. [30] evaluated calibration procedures based on a single posture executed in a self-placement condition, as well as the condition of passive placement by an operator. Intervention of an external operator has been revealed to significantly increase the accuracy of the calibration process. While this issue has been examined in a sports context, such as skiing [40], to the best of the authors knowledge, cycling has not been the subject of particular interest with regard to the development of dedicated calibration methods. Indeed, despite promising technological developments enabling lower limb motion tracking during pedaling motion based on IMUs, no study has addressed the sensor-to-body misalignment [41,42,43]. However, cycling appears to be propitious for setting up embedded calibration tasks using the implicit constraint at the foot-pedal interface, which in turn may improve the accuracy of 3D knee joint angles during the pedaling motion. 

With regard to the importance of the knee joint in cycling, the aim of this study was to propose a novel calibration method for assessing knee joint kinematics during cycling using accelerometer and gyroscope data, without requiring any external operator. A comparison of this new method to conventional ones was proposed. To this end, the accuracy of the calibration process was evaluated in terms of body segment orientation and corresponding 3D joint angles during pedaling motion, with respect to conventional ISB model based on bony landmarks [44,45].

## 2. Materials and Methods

### 2.1. Participants

Fourteen healthy recreational cyclists took part in the study. All participants, 10 males and 4 females (21 ± 3 years, 172.42 ± 8.92 cm, 66.86 ± 16.54 kg), signed an informed consent form in agreement with the local ethical committee and conducted in accordance with the 1975 declaration of Helsinki.

### 2.2. Experimental Set Up

#### 2.2.1. Motion Capture Equipment

Kinematic data were simultaneously recorded using an inertial motion capture system and an optoelectronic motion capture system. The inertial system (MTw, Xsens Technologies, Enschede, The Netherlands) had small dimensions (34.5 × 57.8 × 14.5 mm); it was a low power and low weight (27 g) device that consisted of 3D accelerometers and 3D gyroscopes. Accelerometer and gyroscope data were sampled at the same frequency of 1800 Hz with a full-scale set at ±16 g and ±1200°/s, respectively. Analog inertial data were low-pass filtered (120 Hz for the accelerometers and 140 Hz for the gyroscopes) and transmitted via a wireless Bluetooth connection with a sampling frequency of 75 Hz. The IMUs were firmly attached on the thigh and shank of the right leg with custom elastic bands. The IMU on the thigh (TH) was placed in the middle of the body segment aligning the Y-IMU axis to the long axis of the thigh, with the Z-IMU axis pointing laterally. The IMU on the shank (SH) was placed over the bulge of the gastrocnemius on the lateral face, aligning the Y-IMU axis to the long axis of the shank, with the Z-IMU axis pointing laterally. The estimation of the orientation of the IMU sensors was computed by combining raw data from the gyroscopes and accelerometers through a Madgwick filter as in [46]. 

**Figure 1:**
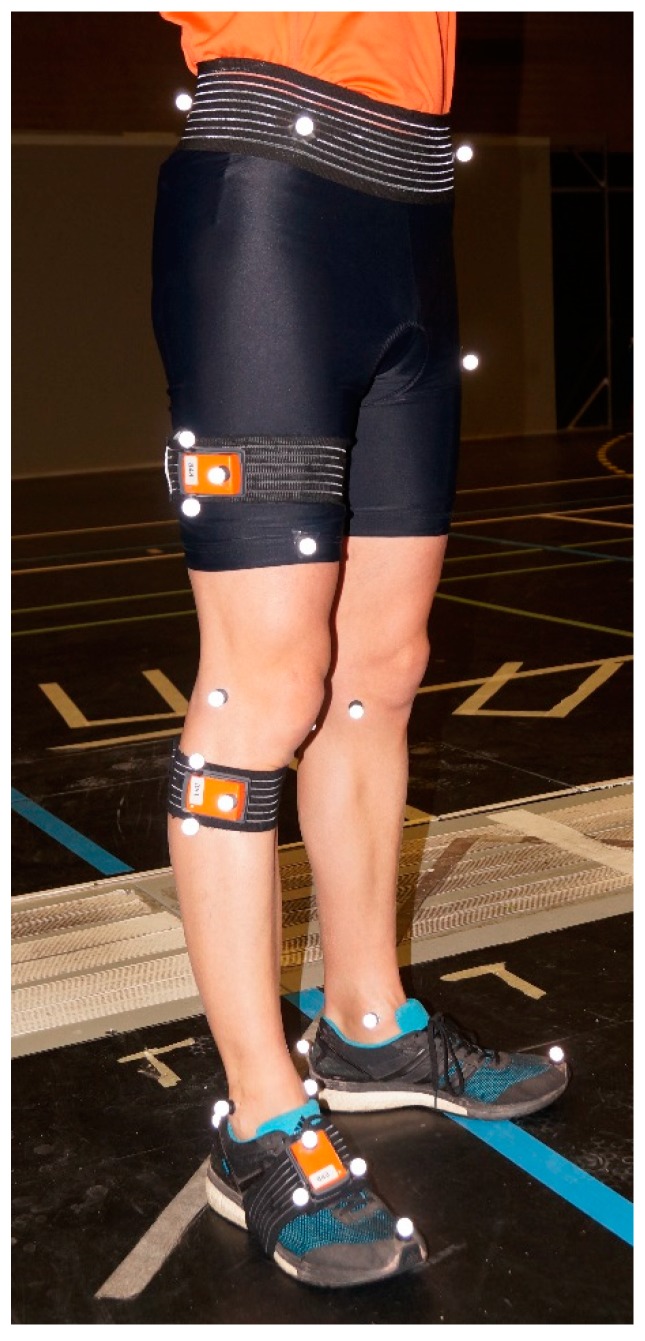
Subject equipped with an inertial measurement unit (IMU) and reflective markers.

The optoelectronic motion capture system was set-up with 12 VICON cameras (Oxford Metrics, Inc., Oxford, UK) operating at 100 Hz. Participants were equipped with a full-body set of 43 markers placed on anatomical landmarks to calculate the anatomical rotations following the International Society of Biomechanics (ISB) recommendations [45]. To evaluate the real orientation of the inertial sensors, three markers were fixed on each IMU to define a cluster. Orientations of the clusters were computed based on optical motion capture system [15,31,41,47,48] (Figure 1). The 3D coordinates were smoothed using a second-order Butterworth low-pass filter with a cut-off frequency of 10 Hz as described in [49].

#### 2.2.2. Definition of the Coordinate Systems

In biomechanics, joint angles are calculated by comparing the relative orientations between the anatomical frames established for adjacent body segments that are proximal and distal to the joint of interest. Using optical systems, the anatomical frames are defined based on the anatomical landmarks. Owing to different conventions for defining anatomical frames based on IMU measurements, the calculated anatomical frame was thus different from the one obtained by the optical system, for any body segment. Anatomical joint angles (e.g., flexion/extension, abduction/adduction, and internal/external rotation) can be determined from the body segment anatomical frames through a Cardan sequence of rotation [50].

**Figure 2:**
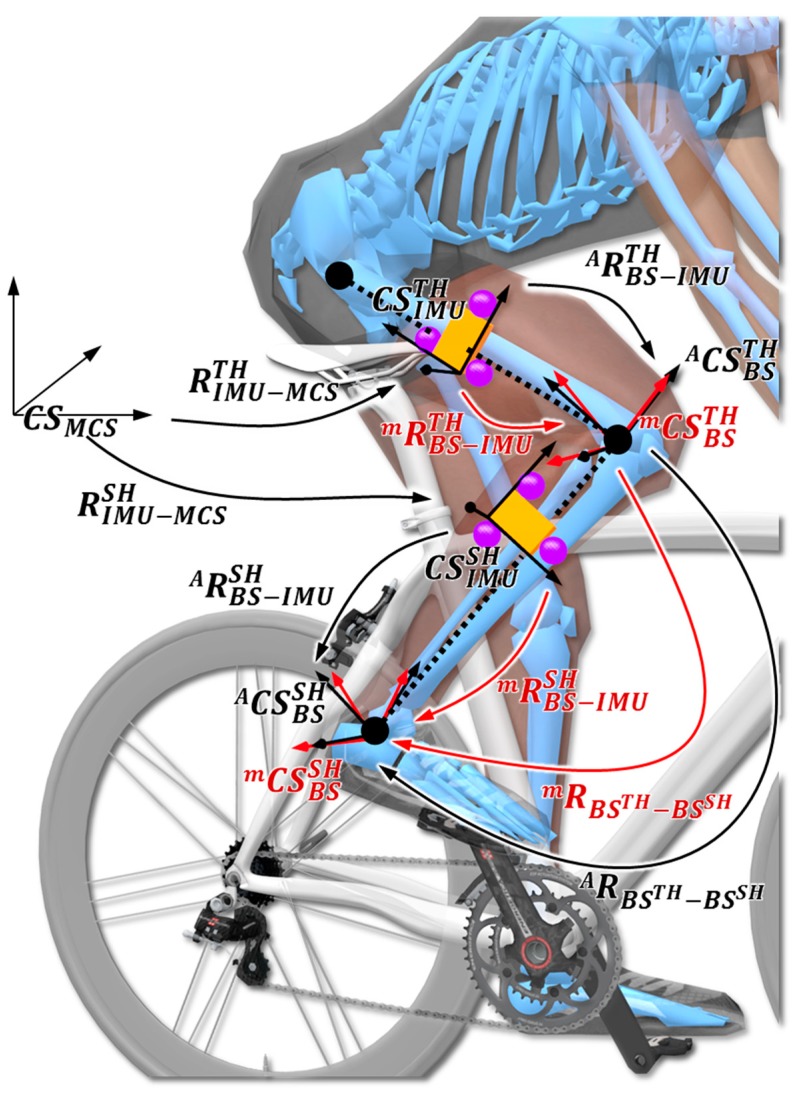
Coordinate systems definition and the rigid transformations between them. Orange box represents the IMU sensors, whereas the purple spheres correspond to markers fixed on the IMU (CASBSSH and CASBSTH) referring to the anatomical body segment coordinate systems (black vectors) and technical body segment frame (red vectors) for the thigh and the shank (CmSBSSH and CmSBSTH). $CS_{IMU}^{TH}$ and $CS_{IMU}^{SH}\;$ represent the IMU coordinate systems for the thigh and the shank. $CS_{J}$ defines the knee joint coordinates following the recommendation of the International Society of Biomechanics [45].

Body segment frames, IMU frames, and the knee joint frame are depicted in Figure 2. The anatomical body segment coordinate systems for the thigh and the shank CASBSTH=[XABSTHYABSTHZABSTH] and CASBSSH=[XABSSHYABSSHZABSSH] respectively, are obtained using optoelectronic motion capture system and ISB model based on anatomical landmarks. Moreover, the IMU coordinate systems for the thigh and the shank are defined as $CS_{IMU}^{TH}=\left[\begin{array}{ccc} X_{IMU}^{TH} & Y_{IMU}^{TH} & Z_{IMU}^{TH} \end{array}\right]$ and $CS_{IMU}^{SH}=\left[\begin{array}{ccc} X_{IMU}^{SH} & Y_{IMU}^{SH} & Z_{IMU}^{SH} \end{array}\right]$, respectively. Based on anatomical frames computed from anatomical landmarks and optoelectronic motion capture, the knee joint coordinate system ${\mathrm{CS}}_{J}=\left[\begin{array}{ccc} X_{J} & Y_{J} & Z_{J} \end{array}\right]$ is defined following the recommendations of the ISB [44,45]. Thus, flexion/extension corresponds to a rotation about the *Z*-axis of the thigh coordinate system $Z_{J}$ = $Z_{\mathrm{BS}}^{\mathrm{TH}}$, the internal/external rotation is defined as a rotation about the *Y*-axis of the thigh coordinate system $Y_{J}=Y_{\mathrm{BS}}^{\mathrm{SH}}$, and abduction/adduction is defined as a rotation about the floating axis. From the markers rigidly attached to the IMU, clusters were defined for the thigh and the shank. The cluster coordinate systems are defined as $CS_{CLU}^{TH}=\left[\begin{array}{ccc} X_{CLU}^{TH} & Y_{CLU}^{TH} & Z_{CLU}^{TH} \end{array}\right]$ and $CS_{CLU}^{SH}=\left[\begin{array}{ccc} X_{CLU}^{SH} & Y_{CLU}^{SH} & Z_{CLU}^{SH} \end{array}\right]$, respectively. Finally, $CS_{MCS}=\left[\begin{array}{ccc} X_{MCS} & Y_{MCS} & Z_{MCS} \end{array}\right]$ corresponds to the global coordinate system of motion capture systems. The body segment frames estimated using the IMU are slightly different from the anatomical body segment frame CASBSTH and CASBSSH that are commonly calculated from bony landmarks or X-rays. Since anatomical frames cannot be directly expressed using IMU measurements, technical body segment frames are commonly used as an estimate of anatomical frames, sometimes called technical–anatomical frames [51]. Since such technical frames depend on the sensor-to-segment calibration method, in the remainder of the paper, we use CmSBSTH and CmSBSSH to indicate the technical body segment frames of the thigh and shank estimated from IMU measurements for a given calibration method m. Details related to the identification of unit vectors used to define these technical frames are considered in Section 2.3.2.

### 2.3. IMU-to-Body Alignment Methods

#### 2.3.1. Calibration Tasks

Participants attended one measurement session divided into two types of calibration task. Firstly, a conventional calibration procedure where each participant randomly performed five typical calibration tasks was conducted. The tasks used in this first calibration procedure are widely used in the literature on sensor-to-segment alignment [18,30,31,34]. Each participant randomly performed the following calibration tasks, according to the instructions given by the operator at the beginning of calibration step:

- Standing up posture (SU): Static standing upright posture with feet apart in line with the hip and knee stretched. In this posture, the longitudinal axis of the thigh and shank are assumed to be vertical. The lower limb posture is similar to the one used during the T-pose or N-pose [30].
- Lying down (LD): Static lying face down posture. Hence, the anteroposterior axis of the segments is assumed to be aligned with the vertical axis. Hands are placed between the ground and chin.
- Knee flexion/extension (KFE): Dynamic task with the participant performing four knee flexions/extensions from about 10° to 90° of flexion, in a single leg up-right posture. Participants were asked to avoid any thigh movement. This task allowed estimation of the knee flexion axis.
- Hip abduction/adduction (HAA): Dynamic task with the participant performing four hip abductions/adductions in a single leg up-right posture. Participants were asked to avoid hip external rotations with the foot pointing forward. This task allowed estimation of the anteroposterior axis of the thigh.

During each repetition, the IMU and optoelectronic motion capture outputs were acquired over five seconds during static tasks, and over four repetitions during dynamic tasks.

During a second calibration step, participants performed a pedaling task (P) for 2 min at a mean cadence of 80 rpm. They performed each condition at a workload of 100 W, on a stationary SRM Indoor trainer (Schoberer Rad Meßtechnik, Jülich, Germany). Power output was measured using an SRM system (Schoberer Rad Meßtechnik, Science version, Germany) calibrated according to the manufacturer’s recommendations. Data were recorded throughout the trials. All tasks are depicted in Table 1. Each task allowed the identification of one or two body segment axis orientations in relation to the IMU frames.

#### 2.3.2. Calibration Methods

Based on the calibration tasks, calibration matrices were obtained by combining pairs of static or dynamic tasks. Four calibration methods are presented in this article and they correspond to the task combinations depicted in Table 2. Each method systematically required the SU task. The anatomical calibration method was defined as the reference method. Other methods employed in this study were called functional methods in contrast to the anatomical method. Given each calibration method, the calculation of the base vectors of the body segment frames is detailed in the following paragraphs.

Static calibration procedure (S)—This calibration method combined the SU posture with LD tasks. 

**Figure 3:**
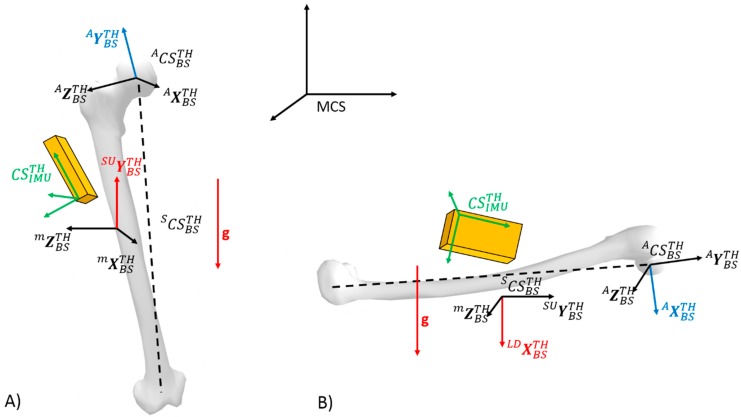
Illustration of the static calibration procedure for the thigh including all considered coordinate systems. The red vectors represent the gravity vector and the axis of the technical body-segment frame which components have to be estimated with respect to the IMU frame for a given task. More specifically, components of ${}^{SU}Y_{BS-IMU}^{TH}$ are estimated from SU task (**A**) whereas components of ${}^{LD}Y_{BS-IMU}^{TH}$ estimated from LD task (**B**). The same procedure can be developed for the shank.

The idea of the procedure is to obtain the orientation the local technical body-segment coordinate frame with respect to the local IMU frame. In other words, a rotation matrix containing the three unit vectors of each segment, expressed in the IMU coordinate system, was obtained. The methodology relies on the following steps, where the components of each body segment axis (technical frame) had to be computed based on accelerometer signals during static SU and LD postures. As an example, concerning the static calibration procedure for the thigh, the Figure 3 depicts the axis of the technical body-segment frame for which components have to be estimated with respect to the IMU frame for a given task (SU or LD). 

The *Y*-axis of technical frames could be found by measuring the direction of gravity during the SU posture. Indeed, for such a posture, the accelerometer of the IMU sensors for the thigh and the shank only measure the gravity vector g that corresponds to the components of g with respect to the IMU frame for the thigh $CS_{IMU}^{TH}$, and the shank $CS_{IMU}^{SH}$. In order to obtain the components of the *Y*-axis with respect to the IMU frame, the idea was thus to align the *Y*-axis with an identifiable vector for which the components could be measured using the IMU signals. It was therefore hypothesized that the *Y*-axis of the technical frame was aligned with the vertical (with respect to the global reference frame) gravity vector g. Thus, computation of the component of the *Y*-axis of the technical frames with respect to the IMU frames ($Y_{\mathrm{BS}-\mathrm{IMU}}^{\mathrm{TH}}$ and $Y_{\mathrm{BS}-\mathrm{IMU}}^{\mathrm{SH}\;}$) was obtained from the accelerometer measurement as

(1) $${}^{SU}Y_{BS-IMU}^{TH}{=}^{SU}Y_{BS-IMU}^{SH}=\frac{-g}{\left|g\right|}$$

The same way, during the LD posture, the accelerometers of the thigh and the shank measured the gravity vector g, which enabled us to obtain the components of g in the thigh body segment coordinate system ${}^{S}CS_{BS}^{TH}$, and in the shank body segment coordinate system ${}^{S}CS_{BS}^{SH}$. 

Moreover, for the LD posture, the *X*-axis of the thigh and shank were aligned with the vertical gravity vector g

(2) $${}^{LD}X_{BS-IMU}^{TH}{=}^{LD}X_{BS-IMU}^{SH}=\frac{g}{\left|g\right|}$$

Therefore, the components of $Z_{BS-IMU}^{TH}$ and $Z_{BS-IMU}^{SH\;}$ expressed in the IMU frame of the thigh and shank are obtained as

(3) $${}^{SU-LD}Z_{BS-IMU}^{TH}=\frac{{}^{LD}X_{BS-IMU}^{TH}\times^{SU}Y_{BS-IMU}^{TH}}{\left|{}^{LD}X_{BS-IMU}^{TH}\times^{SU}Y_{BS-IMU}^{TH}\right|}$$

(4) $${}^{SU-LD}Z_{BS-IMU}^{SH}=\frac{{}^{LD}X_{BS-IMU}^{SH}\times^{SU}Y_{BS-IMU}^{SH}}{\left|{}^{LD}X_{BS-IMU}^{SH}\times^{SU}Y_{BS-IMU}^{SH}\right|}$$

In order to obtain the orthogonal frames, $X_{BS-IMU}^{TH}$ and $X_{BS-IMU}^{SH}$ are finally obtained as

(5) $${}^{SU-LD}X_{BS-IMU}^{TH}={\;}^{SU}Y_{BS-IMU}^{TH}\times^{SU-LD}Z_{BS-IMU}^{TH}$$

(6) $${}^{SU-LD}X_{BS-IMU}^{SH}={\;}^{SU}Y_{BS-IMU}^{SH}\times^{SU-LD}Z_{BS-IMU}^{SH}$$

Finally, the rotation matrix between $CS_{IMU}^{TH}$ and ${}^{S}CS_{BS}^{TH}$, as well as the rotation matrix between $CS_{IMU}^{SH}$ and ${}^{S}CS_{BS}^{SH}$, are respectively obtained by grouping the unit vectors as

(7) $${}^{S}R_{BS-IMU}^{TH}=\left[\begin{array}{ccc} {}^{SU-LD}X_{BS-IMU}^{TH} & {}^{SU}Y_{BS-IMU}^{TH} & {}^{SU-LD}Z_{BS-IMU}^{TH} \end{array}\right]$$

(8) $${}^{S}R_{BS-IMU}^{SH}=\left[\begin{array}{ccc} {}^{SU-LD}X_{BS-IMU}^{SH} & {}^{SU}Y_{BS-IMU}^{SH} & {}^{SU-LD}Z_{BS-IMU}^{SH} \end{array}\right].$$

${}^{S}R_{BS-IMU}^{TH}$ and ${}^{S}R_{BS-IMU}^{SH}$ correspond to the calibration matrices between the IMU frame and technical body segment frame, respectively, for the thigh and for the shank, obtained by the static method.

Mixed calibration procedure (M)—In this procedure, orientations of the body segment frames were obtained by combining the SU static task, the dynamic KFE task, and the dynamic HAA task. Thus, components of ${}^{SU}Y_{BS-IMU}^{SH}$ in the IMU coordinate system of the shank were obtained following the SU task, and were computed as shown in Equation (1). In the same way, components of ${}^{SU}Y_{BS-IMU}^{TH}$ were obtained in the IMU coordinate system of the thigh according to Equation (1). 

Based on the angular velocities measured by the gyroscopes during the KFE task, the mean rotation vector was calculated in the shank IMU frame as described in [52]. Indeed, in this process, the direction of the joint axis of rotation ${}^{KFE}Z_{BS-IMU}^{SH}$ was assumed to coincide with the direction of the mean angular velocity vector expressed in the IMU coordinate system and was determined from the orientation of the angular rate during flexion ($\omega_{FLEX}$) or during extension ($\omega_{EXT}$). The instantaneous direction of angular rate vector N was thus calculated as the unit vector

(9) $$N=\frac{\omega_{FLEX}}{\left|\omega_{FLEX}\right|}\;\mathrm{during}\;\mathrm{flexion}\;\mathrm{or}\;\;\;N=\frac{-\omega_{EXT}}{\left|\omega_{EXT}\right|}\;\mathrm{during}\;\mathrm{extension}$$

The mean N vector was then obtained by averaging over the duration of the movement. Note that the average estimation may not be a unit vector, which should be normalized to be the final ${}^{KFE}Z_{BS-IMU}^{SH}$ estimate. In order to obtain a more reliable rotation axis, it is necessary to limit the angular rate ranges when estimating the rotation axis [33,35,38]. To eliminate noise from the direction of angular rate estimates, only angular rate components exceeding a threshold set at a value of 20% of the maximal angular rate during each functional task were considered.

Moreover, $X_{BS-IMU}^{SH}$ axis is computed from the cross product

(10) $${}^{SU-KFE}X_{BS-IMU}^{SH}=\;\frac{{}^{SU}Y_{BS-IMU}^{SH}\times^{KFE}Z_{BS-IMU}^{SH}}{\left|{}^{SU}Y_{BS-IMU}^{SH}\times^{KFE}Z_{BS-IMU}^{SH}\right|}$$

In order to obtain an orthogonal frame, $Z_{BS-IMU}^{SH}$ is computed again as

(11) $${}^{SU-KFE}Z_{BS-IMU}^{SH}={\;}^{SU-KFE}X_{BS-IMU}^{SH}\times^{SU}Y_{BS-IMU}^{SH}$$

Finally, the rotation matrix ${}^{M}R_{BS-IMU}^{SH}$ between $CS_{IMU}^{SH}$ and ${}^{M}CS_{BS}^{SH}$ is obtained by grouping the three unit vectors

(12) $${}^{M}R_{BS-IMU}^{SH}=\left[\begin{array}{ccc} {}^{SU-KFE}X_{BS-IMU}^{SH} & {}^{SU}Y_{BS-IMU}^{SH} & {}^{SU-KFE}Z_{BS-IMU}^{SH} \end{array}\right]$$

Based on the angular velocities measured by the gyroscopes during the HAA task, the mean rotation vector was calculated in the thigh IMU frame as in [52]. Indeed, in this process, the direction of the joint axis of rotation ${}^{HAA}X_{BS-IMU}^{TH}$ was assumed to coincide with the direction of the mean angular velocity vector expressed in the IMU coordinate system and was determined from the orientation of the angular rate during abduction ($\omega_{ABD}$) or during adduction ($\omega_{ADD}$). The instantaneous direction of angular rate vector N was thus calculated as the unit vector

(13) $$N=\frac{\omega_{ABD}}{\left|\omega_{ABD}\right|}\;\mathrm{during}\;\mathrm{abduction}\;\mathrm{or}\;\;\;N=\frac{-\omega_{ADD}}{\left|\omega_{ADD}\right|}\;\mathrm{during}\;\mathrm{adduction}$$

The mean N vector was then obtained by averaging over the duration of the movement. Note that the average estimation may not be a unit vector, which should be normalized to be the final ${}^{HAA}X_{BS-IMU}^{TH}$ estimate. To eliminate noise from the direction of angular rate estimates, only angular rate components exceeding a threshold set at a value of 20% of the maximal angular rate during each functional task were considered.

Moreover, $Z_{BS-IMU}^{TH}$ axis is computed from the cross product

(14) $${}^{SU-HAA}Z_{BS-IMU}^{TH}=\;\frac{{}^{HAA}X_{BS-IMU}^{TH}\times^{SU}Y_{BS-IMU}^{TH}}{\left|{}^{HAA}X_{BS-IMU}^{TH}\times^{SU}Y_{BS-IMU}^{TH}\right|}$$

In order to obtain an orthogonal frame $X_{BS-IMU}^{TH}$ is computed again in the IMU frame as

(15) $${}^{SU-HAA}X_{BS-IMU}^{TH}{=}^{SU}Y_{BS-IMU}^{TH}\times^{SU-HAA}Z_{BS-IMU}^{TH}.$$

Finally, the rotation matrix ${}^{M}R_{BS-IMU}^{TH}$ between $CS_{IMU}^{TH}$ and ${}^{M}CS_{BS}^{TH}$ is obtained by grouping the three unit vectors

(16) $${}^{M}R_{BS-IMU}^{TH}=\left[\begin{array}{ccc} {}^{SU-HAA}X_{BS-IMU}^{TH} & {}^{SU}Y_{BS-IMU}^{TH} & {}^{SU-KFE}Z_{BS-IMU}^{TH} \end{array}\right]$$

Cycling calibration procedure (C)—In this procedure, orientations of the body segment frames were obtained by coupling of the SU and P tasks. As for methods previously developed, components of ${}^{SU}Y_{\mathrm{BS}-\mathrm{IMU}}^{\mathrm{TH}}$ and ${}^{\mathrm{SU}}Y_{\mathrm{BS}-\mathrm{IMU}}^{\mathrm{SH}}$ in the IMU coordinate system of the thigh and shank were obtained following the SU posture, and computed as shown in Equation (1). Similar to the mixed method and based on the angular velocities measured by the gyroscopes during the pedaling motion, the mean rotation vector was calculated in the IMU frame of the thigh and shank. Assuming that the pedaling motion occurs primarily in the sagittal plane, the mean rotation vector of the shank and thigh IMU is orthogonal to this plane, and it is aligned to $Z_{\mathrm{BS}}^{\mathrm{TH}}$ and $Z_{\mathrm{BS}}^{\mathrm{SH}}$. Hence, ${}^{P}Z_{\mathrm{BS}-\mathrm{IMU}}^{\mathrm{TH}}$ and ${}^{P}Z_{\mathrm{BS}-\mathrm{IMU}}^{\mathrm{SH}}$ expressed in the local IMU coordinate system can be determined from the orientation of the angular rate vector using the same computation for ${}^{\mathrm{KFE}}Z_{\mathrm{BS}-\mathrm{IMU}}^{\mathrm{SH}}$. Moreover, $X_{\mathrm{BS}-\mathrm{IMU}}^{\mathrm{TH}}$ and $X_{\mathrm{BS}-\mathrm{IMU}}^{\mathrm{SH}}$ are computed from the cross product

(17) $${}^{SU-P}X_{BS-IMU}^{TH}=\;\frac{{}^{SU}Y_{BS-IMU}^{TH}\times^{P}Z_{BS-IMU}^{TH}}{\left|{}^{SU}Y_{BS-IMU}^{TH}\times^{P}Z_{BS-IMU}^{TH}\right|}$$

(18) $${}^{SU-P}X_{BS-IMU}^{SH}=\;\frac{{}^{SU}Y_{BS-IMU}^{SH}\times^{P}Z_{BS-IMU}^{SH}}{\left|{}^{SU}Y_{BS-IMU}^{SH}\times^{P}Z_{BS-IMU}^{SH}\right|}$$

As for previous methods, $Z_{BS-IMU}^{TH}$ and $Z_{BS-IMU}^{SH}$ were computed using the cross product with respect to the orthogonality of the frames. Finally, the rotation matrix ${}^{C}R_{BS-IMU}^{TH}$ between $CS_{IMU}^{TH}$ and ${}^{C}CS_{BS}^{TH}$, as well as the rotation matrix ${}^{C}R_{BS-IMU}^{SH}$ between $CS_{IMU}^{SH}$ and ${}^{C}CS_{BS}^{SH}$, were respectively obtained by grouping the unit vectors as

(19) $${}^{C}R_{BS-IMU}^{TH}=\left[\begin{array}{ccc} {}^{SU-P}X_{BS-IMU}^{TH} & {}^{SU}Y_{BS-IMU}^{TH} & {}^{SU-P}Z_{BS-IMU}^{TH} \end{array}\right]$$

(20) $${}^{C}R_{BS-IMU}^{SH}=\left[\begin{array}{ccc} {}^{SU-P}X_{BS-IMU}^{SH} & {}^{SU}Y_{BS-IMU}^{SH} & {}^{SU-P}Z_{BS-IMU}^{SH} \end{array}\right]$$

Reference anatomical calibration procedure (A)—This method combined both the SU and P tasks. Starting on the one hand from CASBSTH and CASBSSH (defining anatomical frames obtained from optoelectronic motion capture system and bony landmarks-based ISB model) and on the other hand from $CS_{CLU}^{TH}$ and $CS_{CLU}^{SH}$ (defining cluster frames of the thigh obtained from optoelectronic motion capture system) during the SU task, the longitudinal *Y*-axis of anatomical frames for the thigh and the shank in relation to the cluster frames are obtained as

(21) YABS−CLUTH=RCLU−MCSTH*YABS−MCSTH

(22) YABS−CLUSH=RCLU−MCSSH*YABS−MCSSH

where $R_{CLU-MCS}^{TH}$ and $R_{CLU-MCS}^{SH}$ represents the orientation of the cluster frames in relation to the global coordinate system of the motion capture system, for the thigh and the shank respectively. Moreover, in order to obtain the medio-lateral axis ZABSTH and ZABS−CLUSH, the functional method known as the “Symmetrical Axis of Rotation Assessment” SARA [53], was used during the pedaling task.

The floating axes XABSTH and XABSSH were obtained as

(23) $${}^{A}X_{BS-CLU}^{TH}=\;\frac{{}^{A}Y_{BS-CLU}^{TH}\times^{A}Z_{BS-CLU}^{TH}}{\left|{}^{A}Y_{BS-CLU}^{TH}\times^{A}Z_{BS-CLU}^{TH}\right|}$$

(24) $${}^{A}X_{BS-CLU}^{SH}=\;\frac{{}^{A}Y_{BS-CLU}^{SH}\times^{A}Z_{BS-CLU}^{SH}}{\left|{}^{A}Y_{BS-CLU}^{SH}\times^{A}Z_{BS-CLU}^{SH}\right|}$$

Furthermore, ${}^{A}Z_{BS-CLU}^{TH}$ and ${}^{A}Z_{BS-CLU}^{SH}$ was computed as

(25) $${}^{A}Z_{BS-CLU}^{TH}={\;}^{A}X_{BS-CLU}^{TH}\times^{A}Y_{BS-CLU}^{TH}$$

(26) $${}^{A}Z_{BS-CLU}^{SH}={\;}^{A}X_{BS-CLU}^{SH}\times^{A}Y_{BS-CLU}^{SH}$$

Finally, rotation matrices between anatomical frames and cluster frames were obtained as

(27) $${}^{A}R_{BS-CLU}^{TH}=\left[\begin{array}{ccc} {}^{A}X_{BS-CLU}^{TH} & {}^{A}Y_{BS-\mathrm{CLU}}^{TH} & {}^{A}Z_{BS-CLU}^{TH} \end{array}\right]$$

(28) $${}^{A}R_{BS-CLU}^{SH}=\left[\begin{array}{ccc} {}^{A}X_{BS-CLU}^{SH} & {}^{A}Y_{BS-CLU}^{SH} & {}^{A}Z_{BS-CLU}^{SH} \end{array}\right]$$

Due to the imperfect placement of the markers on the sensor, the cluster frame is not perfectly aligned with that of the IMU chip. To correct this misalignment, we used the alignment method used by [16] which uses angular velocities to align local coordinate systems (IMU-based and optical-based). As underlined by [54], the alignment error from the methods based on angular velocities was significantly lower than for the other conventional methods. For each subject, the misalignment was quantified around the axes of the cluster and was less than 1° around the *X*-axis, 26° around the *Y*-axis and 2° around the *Z*-axis. The literature (e.g., [16,54,55]) showed that the method proposed by de Vries enables an alignment with an error lower than 1°.

All calibration matrices and corresponding combinations of tasks are summarized in Table 2.

### 2.4. Joint Angles Computation

The joint coordinate system ($CS_{J}$) was used to describe the knee joint angles [45] calculated as the difference of body segment frames orientation during the cycling task. Thus, knee joint angles were expressed with respect to each method. The anatomical calibration method allowed computation of the orientation of the anatomical body frame attached to the thigh ${}^{A}R_{BS-MCS}^{TH}$ and shank ${}^{A}R_{BS-MCS}^{SH}$. The static, mixed, and cycling methods are functional calibration methods that enable assessment of the orientation of three technical frames, each one being associated to the corresponding method for the thigh and shank. The static method was used to estimate the calibration matrices of the technical body segment frames ${}^{S}R_{BS-MCS}^{TH}$ and ${}^{S}R_{BS-MCS}^{SH}$. Similarly, the mixed and cycling methods were used to estimate ${}^{M}R_{BS-MCS}^{TH}$, ${}^{M}R_{BS-MCS}^{SH}$, and ${}^{C}R_{BS-MCS}^{TH}$, ${}^{C}R_{BS-MCS}^{SH}$, respectively. For each method, the knee orientation was expressed through the following rotation matrix

(29) $${}^{m}R_{J}={\;}^{m}R_{BS^{TH}-BS^{SH}}={\left({}^{S}R_{BS-MCS}^{TH}\right)}^{T}\;.^{S}R_{BS-MCS}^{SH}$$

where m denotes the method employed. Finally, using a ZYX rotation sequence as described in [45], the Euler angles were obtained for each subject based on the reference method as (${}^{A}\theta_{x}$, ${}^{A}\theta_{y}$, ${}^{A}\theta_{z}$), and for the method as (${}^{m}\theta_{x}$, ${}^{m}\theta_{y}$, ${}^{m}\theta_{z}$) during the cycling task. 

### 2.5. Data Analysis

The estimation of the orientation of the IMU sensors was computed by combining raw data from the gyroscopes and accelerometers through a Madgwick filter [46]. More specifically, the filter gain β was adjusted at a value of 0.055 rad/s according to a trial-and-error procedure. Precautions were taken as regards to the initial orientation state and the duration of each trial. We indeed took care to coincide the beginning of each trial with the initial orientation state, which is a factor in favor of a lower drift. Finally, short duration tasks (lower than 30 s) were considered in the present study, in order to limit this phenomenon (for all subjects, joint angle error was computed with root mean square error only during the nine first seconds in order to obtain a minimum of ten pedaling cycles). 

The anatomical frames based on the IMU system and the frames based on the optical system are most likely different due to the different protocols used in determining the transformation matrices between the sensor or cluster frames and the camera-based body-segment frame. This could lead to differences in the joint angle values [30,39]. The objectives of the data analysis were: first, to evaluate the accuracy of each calibration method by comparing the alignment of the body segment frames obtained by the inertial system and the frames obtained by a reference optical system; and second, to assess the differences in knee joint angular displacements between the IMU-based calculations and the anatomical method. Data analysis was carried out using the MATLAB software (Mathworks, Natick, MA, USA). 

#### 2.5.1. Alignment Error between the IMU and the Body Segment Frames

The accuracy of the calibration methods was computed as the alignment error obtained from computing of the rotation matrix between the orientation of the anatomical frame and the technical frame orientation of the functional method of interest. 

The rotation matrix of the alignment error ${}^{m}E_{IMU-BS}$ was calculated following Equation (30).

(30) $${}^{m}E_{IMU-BS}^{s}={\left({}^{A}R_{BS-IMU}^{s}\right)}^{T}\;.{\;}^{m}R_{BS-IMU}^{s}$$

where m corresponds to the method (e.g., C) and s corresponds to the considered body segment (e.g., SH). Furthermore, the amplitude of the error that corresponded to the accuracy of the alignment method was calculated using Equation (31) as in [33].

(31) $${}^{m}\theta_{IMU-BS}^{s}=\cos^{-1}\left(\frac{Tr\left({}^{m}E_{IMU-BS}^{s}\right)-1}{2}\right)$$

where ${}^{m}\theta_{IMU-BS}^{s}$ refers to the angle of method m and segment s. For a given calibration method, the mean error and standard deviation were calculated over the individual errors ${}^{m}\theta_{IMU-BS}^{s}$.

Moreover, angles between the X, Y, and Z axes from the IMU frame and body segment frame were calculated as the smallest angles between the base vectors. Indeed, ${}^{m}\theta_{IMU-BS}^{s}$ only gives an overall error of orientation. However, the alignment error could be affected by a given method only around a specific body segment axis [33]. To this aim, accuracy is expressed as Euler angles from the rotation matrix ${}^{m}E_{IMU-BS}^{s}$, with the Euler sequence ‘YZX’. The alignment errors around each anatomical axis ${}^{m}\theta_{x}^{s}$, ${}^{m}\theta_{y}^{s}$, ${}^{m}\theta_{z}^{s}$ were computed as

(32) mθxs= tan−1(−YmBS_IMUs·ZABS_IMUsYmBS_IMUs·YABS_IMUs)

(33) mθzs=sin−1(YmBS_IMUs·XABS_IMUs)

(34) mθys= tan−1(−ZmBS_IMUs·XABS_IMUsXmBS_IMUs·XABS_IMUs)

For each method m (Static (S), Mixed (M) and Cycling (C)), the technical frame was obtained using the same SU task leading to the same Ym axis

(35) YSBS_IMUs= YMBS_IMUs= YCBS_IMUs=YSUBS_IMUs

The alignment errors ${}^{m}\theta_{X}^{s}$ et ${}^{m}\theta_{Z}^{s}$ (around *X*-axis and *Z*-axis respectively) were constant across methods since they depend only on Ym and XA, YA, ZA that are identical for all methods. Alignment errors around *X*-axis and *Z*-axis of the anatomical frames ${}^{m}\theta_{x}^{s}$ and ${}^{m}\theta_{z}^{s}$ were finally computed as

(36) SθXs=MθXs=CθXs= tan−1(−YSUBS_IMUs·ZABS_IMUsYSUBS_IMUs·YABS_IMUs)

(37) SθZs=MθZs=CθZs=sin−1(YSUBS_IMUs·XABS_IMUs)

#### 2.5.2. 3D Knee Joint Angle Accuracy

Knee joint angles estimated from the functional methods were compared to the reference angles computed from the anatomical frames based on the optoelectronic motion capture system. Differences were quantified by calculation of the root mean squared error (RMSE) between the waveforms. Thus, the error was computed for each angle between the reference angle (${}^{A}\theta_{x}$, ${}^{A}\theta_{y}$, ${}^{A}\theta_{z}$) and joint angle obtained by the functional methods (${}^{m}\theta_{x}$, ${}^{m}\theta_{y}$, ${}^{m}\theta_{z}$), with the computation of the root mean square error.

### 2.6. Statistical Analysis

Descriptive statistics, including mean and standard deviation, were determined for each outcome measure ${}^{m}\theta_{IMU-BS}$, ${}^{m}\theta_{X}^{s}$, ${}^{m}\theta_{Y}^{s}$, ${}^{m}\theta_{Z}^{s}$ and RMSE of the 3D knee angles. These statistics were calculated for each participant as an average of all repetitions before being averaged across all the participants. For each method and variable, the normal distribution was tested using the Shapiro–Wilk test of normality. Assumption of homogeneity of variance was evaluated with the Levene’s test. A Welch ANOVA was used to compare the means of different methods. The Welch’s test was performed for heterogeneity of variance. When a significant method effect was found, pairwise comparisons were performed using multiple comparisons with the Bonferroni correction. The level of significance was set to 0.05 for all statistical tests. The statistical analysis was performed using the R language.

## 3. Results

### 3.1. Accuracy of the Segment Orientation

**Figure 4:**
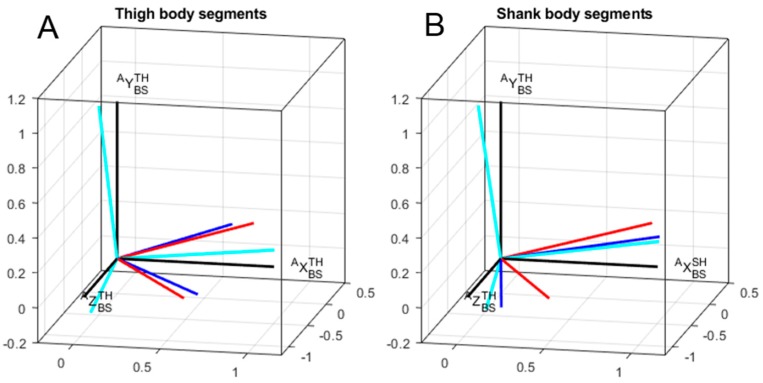
A typical example results for one participant concerning technical frames orientation for thigh (**A**) and shank (**B**) with respect to corresponding anatomical frames. Red: static method; blue: mixed method; cyan: cycling method; black: anatomical method.

Figure 4 depicts a typical result for one participant concerning technical frames orientation for thigh and shank with respect to corresponding anatomical frames. The mean and standard deviation of errors represented as differences between the anatomical frame and technical frames are shown in Table 3. Differences between methods are reported only for the total angle and around the *Y*-axis. By construction of the calibration matrices, the alignment errors around the *X*-axis and *Z*-axis were constant across methods. Indeed, functional calibration matrices shared the same longitudinal axes obtained from the SU task. Differences between methods resulted from a rotation around this *Y*-axis. 

No differences were quantified for the static versus mixed methods with respect to the global orientation, neither for the thigh (*p* = 0.78) nor for the shank (*p* = 0.11). The cycling method did not reveal any significant differences in the global orientation concerning the shank, neither compared to the static approach (*p* = 0.07) nor compared to the mixed method (*p* = 0.45). However, significant lower errors were obtained concerning the thigh for the cycling method (10.9 ± 1.6°) in comparison to both the static method (20.0 ± 6.6°, *p* = 0.003) and the mixed method (17.4 ± 8.4°, *p* = 0.001). 

No differences were quantified along the *Y*-axis for the static versus mixed methods, neither for the thigh (*p* = 0.74) nor for the shank (*p* = 0.11). The cycling method did not reveal any significant differences along the *Y*-axis concerning the shank, neither compared to the static approach (*p* = 0.09) nor compared to the mixed method (*p* = 0.55). However, significant lower errors were obtained concerning the thigh for the cycling method (−1.7 ± 2.3°) in comparison with both the static method (−16.2 ± 8.9°, *p* < 0.001) and the mixed method (−19.1 ± 9.3°, *p* < 0.001). 

### 3.2. Effects of the Calibration Methods on 3D Knee Joint Angles

**Figure 5:**
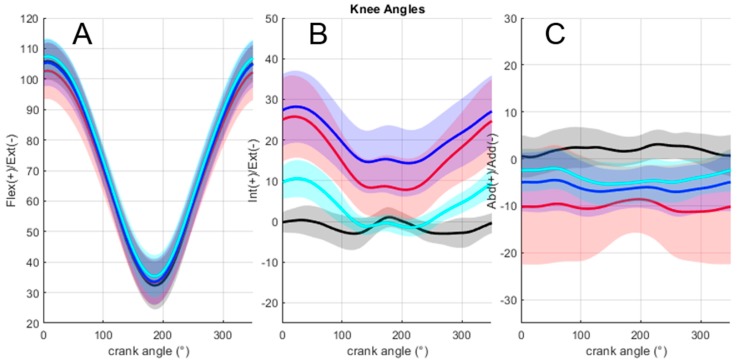
Mean and standard deviation of 3D knee joint angles across the population for all methods. (**A**: flexion/extension; **B**: internal/external rotation; **C**: abduction/adduction). Red: static method; blue: mixed method; cyan: cycling method; black: anatomical method.

Figure 5 depicts mean 3D knee joint angles across the population for all methods. Regarding the influence of calibration methods on the differences in the 3D knee angle values (Table 4), no effect was noticed on the flexion/extension angles in terms of the RMSE. Concerning abduction/adduction, the multiple comparisons showed that the error was significantly lower only for the cycling method in comparison to the static method (11.2 ± 6.6° versus 5.9 ± 2.9°, *p* < 0.05). Concerning the internal/external rotation, the cycling method exhibited a significantly lower error (6.7 ± 1.9°) in comparison to both the static (15.4 ± 5.4°, *p* < 0.01) and mixed methods (18.8 ± 8.0°, *p* < 0.01). 

## 4. Discussion

This study was aimed at developing a novel calibration procedure for the IMU-based kinematic analysis of cycling. In particular, given the major contribution of the knee joint to cycling performance, the proposed methodology was set up for the purpose of obtaining the 3D knee joint angles in ambulatory conditions. The present analysis was restricted to 6 DOF inertial measurements, since bicycle design may induce a non-homogeneous magnetic field [17]. In the frame of this study, the calibration task had to be autonomously feasible, without any external operator or any device. Assessment of the body segment frame based on the IMU, excluding a magnetometer, requires at least two calibration tasks that enable each to identify one body segment axis. It has been hypothesized that a calibration procedure that combined a standing up posture and a pedaling motion could improve the accuracy of the 3D knee joint angles during cycling. To this end, we compared two combinations of conventional functional tasks used in the IMU-based gait analysis to the new calibration method based on pedaling motion by evaluating the accuracy of the body segment axes orientation and the 3D knee joint angle values with respect to ISB model based on bony landmarks.

### 4.1. Differences Between Calibration Methods—Comparisons with the Literature 

In comparison to the static and mixed methods, the cycling method did not reveal any difference in terms of global orientation concerning the shank, but it showed a significantly lower error for the thigh. Comparison of the accuracy of calibration methods with previous works from the literature was not easy given the limited studies focusing on body segment frames orientation concerning the lower limbs. The accuracy of the body segment axes orientation by means of IMU technology has been previously evaluated concerning the upper limbs [33], concerning the lower limbs [38], and concerning both the upper and lower limbs [30]. With regards to the lower limbs orientation, it has been demonstrated that the magnitude of error could vary within a range of 4–11° [33,38]. Recently, Robert-Lachaine et al. [30] reported a longitudinal error close to 5°, either for the upper leg or for the lower leg, around the longitudinal axis, based on a single static N-pose or T-pose using IMU measurements that included a magnetometer. Present results reported errors around the longitudinal axis lower than 2° for the thigh and lower than 10° for the shank with regards to the cycling method, which was consistent with most previous studies that focused on gait analysis [39]. However, errors around the *Y*-axis concerning the static method were close to 15° for both the thigh and shank. These errors are out of the range of errors between 5° and 15° previously reported in [30]. Concerning the mixed method, our results reported an error of 19.1° for the thigh and 10.0° for the shank, with regards to the *Y*-axis. These results were larger than those reported in [33] (range of error between 4.0° and 17.0°). Although this latter study focused on the upper limb, it was one of the few that investigated the errors of the body-to-segment misalignment in terms of base vector orientation during dynamic calibration tasks.

Furthermore, as some perturbations of the body segment orientation may affect joint angle accuracy [39], the effects of each calibration process on the knee joint angle accuracy were tested during a pedaling motion. Concerning the flexion/extension angle, the present results reported RMS errors in a range between 3.74 ± 2.99° (cycling method) and 4.79 ± 3.03° (static method), which was in agreement with most studies in the literature that reported errors within a range of 5–10° during gait [56,57]. Present results were demonstrated to be lower than those previously reported in cycling, with a mean error of 6.41° [41]. Concerning the abduction/adduction and internal/external rotations, the errors were revealed to be larger (ranging from 5.92° to 11.18 and from 6.65° to 18.80°, respectively). Errors for abduction/adduction in the present study were close to the errors reported in [34] (4.0–6.3°) during gait analysis. However, the errors concerning internal/external rotation were larger than the errors obtained in [34], except for the cycling calibration method that induced significantly lower errors for the internal/external rotation (6.65 ± 1.94°) in comparison to the static (15.37 ± 5.38°) and mixed (18.80 ± 8.05°) methods. Moreover, the cycling method significantly reduced the error of the abduction/adduction angle in comparison to the static method (5.92 ± 2.85° and 11.18 ± 6.62°).

While the cycling method clearly reduced the errors for the thigh, the offset errors around the Y−axis of the shank were not significantly different between methods. Even if the cycling method presented the lowest errors, differences between the methods were not significant. Indeed, errors for the shank were similar to the errors obtained for the thigh based on the static method (−16.2° for the thigh versus −14.4° for shank). Similar errors were previously reported for the thigh and shank within a range of errors between 5° and 12°, using single-pose calibrations [30]. Concerning the mixed method, a lower error was obtained for the shank (−10.0°) compared to the thigh (−19°).

### 4.2. Variability of Calibration Tasks 

Differences between methods may derive their origins from various parameters such as the type of tasks performed (and their combination), the body posture adopted during these tasks, the self vs. passive placement for each task, or the ability to isolate some degrees of freedom at the joint. 

Since the analysis of the orientation error of the body segment frames revealed a difference between methods only for the thigh, the potential differences concerning the knee angles might essentially be attributed to the estimated orientation of the thigh. Errors related to the thigh could a priori be explained by the manner in which the tasks are combined with each other. Indeed, each method uses a standing up posture that enables identification of the longitudinal body segment axis. This identification is based on a priori knowledge of the vertical body segment orientation of the lower limbs during such a posture. Indeed, during the SU task, each participant maintained a slightly different posture from one subject to another, which may have induced a specific inclination of the anatomical axes of each body segment with respect to the vertical axis [31]. Thus, for each subject, an individual offset between the anatomical and technical frames is induced, which is preserved for each calibration method. Therefore, differences in the methods may mainly be ascribed to the estimation of the remaining body segment axes.

Previous studies demonstrated that the participant’s self-placement (as opposed to passive placement) could decrease the accuracy of the calibration method [30], which may explain why larger errors were reported in our study. More specifically, the LD task used in the present static method, which is similar to the task proposed in [31], may induce a difficulty in correctly performing such a posture. As previously highlighted in [30], this could partly explain differences between the results in [30] and our static method. In such a method, the anteroposterior axis of the thigh was estimated by the LD task. Thus, despite verbal instructions to maintain the foot in a vertical position, an inter-individual variability may have impacted the estimation of *X*-axis. 

Functional methods used in this paper may also be affected by the execution of the calibration tasks either with regard to the ability to isolate pure rotation axis or with regard to its autonomous achievement [58,59]. For the mixed method, the thigh anteroposterior *X*-axis is estimated by the hip abduction/adduction. Imprecision in identifying the anteroposterior axis of the thigh could result from the difficulty to the subject in isolating this axis in a pure abduction movement. Indeed, the inability to perform a perfect mono-axial movement has been highlighted in [60]. Such a difficulty may be magnified for hip abduction/adduction, which requires a thigh lateral elevation in a unipodal posture. As mentioned in [24], achieving calibration dynamic movement ‘purely’ may be difficult for the subjects. Based only on the instruction given to the subject, hip abduction/adduction was often composed of small unexpected internal/external hip rotation. Even if the motion occurred mainly in the frontal plane, thigh lateral elevation could be a combination of different rotations involving the ipsi- and contralateral femoro-acetabular joint [61]. In such a context, Favre et al. [34] were the first to propose the HAA as a calibration task. Fradet et al. [38] evaluated the accuracy of the *X*-axis orientation of the thigh and reported an error larger than 25° using the HAA task in comparison to the SARA reference method [53]. In order to reduce the errors arising from the estimation of the *X*-axis of the thigh by a dedicated task, another solution lies in the estimation of the *Z*-axis using a specific calibration task that involves the knee joint in a functional task. 

Thus, although knee motion is dominated by the rotation around the flexion/extension axis, movements about the secondary axes also occur, which led some authors to use passive knee flexion–extension calibration movements in a range that minimizes the screw-home effect [34,62,63]. Generally, such a task requires the intervention of an external operator, which is not appropriate in an attempt towards self-calibration. Another solution to eliminate the negative influence of movements about the secondary axes (e.g., internal/external rotations) is to consider kinematic models and constraints [23,26,27,28]. It is reasonable to believe that the pedaling movement makes it possible to properly execute a sagittal knee movement at a given frequency and workload, and it enables limiting of the flexion–extension range of motion of the knee without the help of any operator. In that sense, the pedaling motion occurs mostly in the sagittal plane and it is performed in a closed kinematic chain. For this task, the knee joint can thus be reasonably assumed to be a pure hinge joint about the flexion–extension rotation axis, as fewer deviations can be observed in the frontal and horizontal planes and do not exceed 13.1° [9]. Therefore, the new calibration procedure proposed in this article estimated the medio-lateral axis of the thigh from a cycling task. Although the estimation of knee axis based on mean angular rate may engender some inaccuracy in dynamic calibration methods (standard deviation of angular rate vector orientation close to 19° for cycling methods), it should be noted that cycling method improved the accuracy of knee joint angles. Indeed, the cycling method significantly reduced the error around the longitudinal axis of the thigh, while no differences were reported for the knee flexion/extension angle, whatever the calibration method. Such a behavior has been previously highlighted when applied to gait analysis, showing that the flexion–extension angle seems to be robust to thigh frame disturbance [39]. Our results showed that errors around the longitudinal *Y*-axis of thigh were mainly propagated on the internal/external rotation, but to a lesser extent on the abduction/adduction angle with regards to the pedaling motion. Similar conclusions were previously observed in the gait analysis [39] and they showed that a small error around the *Y*-axis may induce larger errors in the internal/external rotation, with moderate errors in the abduction/adduction.

### 4.3. Limitations and Perspectives 

A limitation of this study lies in the fact that the analysis focused mainly on the accuracy of the body segment orientation and knee joint angles, whereas repeatability was not assessed. Therefore, further investigations should focus on the inter-individual reliability as an important issue regarding IMU-based motion capture that is performed autonomously. 

As exercise intensity is known to affect the pedaling pattern [7,9], it is conceivable that the pedaling intensity may impact the estimation of the body segment axes orientation. Previous works have already demonstrated that functional methods such as knee flexion/extension are sensitive to the load applied to the joint [34,59,64]. Thus, a functional calibration task based on the cycling exercise, enabling movement in a sagittal plane at a controlled workload and imposed cadence, may engender fewer errors in body segment orientations with regards to functional tasks involving a distal segment that is free to move without any external resistance.

Furthermore, it should be kept in mind that soft tissue artefacts (STA) are a potential source of error common to the optoelectronic-based or IMU-based motion capture system. However, it may have a dissimilar effect on the body segment orientation or knee joint angle assessment. Concerning the optoelectronic motion capture applied to cycling, a previous study [65] used static calibrations in the cycling context at the hip flexion/extension extrema (pedal up and pedal down positions). They demonstrated that the error of the thigh orientation decreased from more than 5° for these two pedal positions, and they suggested the STA as an influencing factor. Concerning the IMU-based motion capture, it can be hypothesized that the STA may be a predominant source of error for dynamic calibration tasks, which may decrease the accuracy of the mixed method. However, to our knowledge, except [66], no study has investigated the effects of the STA on dynamic calibration tasks based on both the optoelectronic and IMU systems, which may be an interesting prospect to explore. Therefore, it may be questioned whether lower errors reported for the pedaling task are due to a lower STA when performing movements in the sagittal plane. Another limitation lies in the effect of errors in markers placement on calculated joint angles since a small error in the marker placement may induce noticeable errors in knee joint kinematics, particularly in the frontal and transverse planes [67,68].

Finally, it is important to bear in mind that a systematic bias (potentially inducing an offset) is expected when comparing IMU based joint angles and opto-electronic bony landmarks-based joint angles because of the different technologies and constructions used for the segment coordinate system [24,31,69,70]. To overcome this limitation, the combination of segment coordinate systems based on palpable landmarks and pointing devices with IMUs is an interesting alternative [25], but does not meet the criterion of autonomy required in our study.

Main perspectives on this work relate to long-duration measurements and in-field applicability of our method. As regards to the first point, improvements regarding the complementary filter choice and adjustments may be of interest to better control drift of sensor orientation, which is crucial for in situ analysis and more particularly for prolonged exercise in cycling. As regard to a use of the present method out of the lab, another point of interest relates to the initial orientation of the IMU during outdoor cycling. For the purpose of a validation process which was the main aim of this paper, an initial orientation was set as close as possible to the reference orientation obtained from optoelectonical system, as commonly reported in the literature [15,48]. However, an initial orientation estimation based only on IMU data would require a specific fusion algorithm (e.g., extended Kalman filter) [14]. Nevertheless, in view of our results, and as an attempt to assess the in-field applicability of our method, errors relative to the typical ranges of motion in cycling (80° for flexion/extension, 20° for abduction/adduction, and 23° for internal/external rotation) were 4.6% for flexion/extension, 29.5% for abduction/adduction and 28.9% for internal/external rotation, which seems promising for future application related to screening and performance enhancement in cycling. 

## 5. Conclusions

This study contributes a new functional calibration method devoted to cycling activity, which can easily be used in-field and in an autonomous manner. Using this method, three-dimensional rotations across the knee can be estimated using a pair of shank- and thigh-mounted IMUs. The accuracy of this method was compared to conventional calibration methods. Our results demonstrated that a calibration method based on the completion of a pedaling task combined with a standing posture significantly improved the accuracy of 3D knee joint angles when applied to cycling analysis.

## Figures and Tables

**Table 1:** Illustration of the calibration tasks used for the different calibration methods. Each calibration task allows the identification of at least one unit vector for the thigh or shank body segment frames. SU: Standing up posture; LD: Lying down; HAA: Hip abduction/adduction; KFE: Knee flexion/extension; P: Pedaling.

Task.	SU	LD	HAA	KFE	P
Illustration	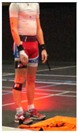	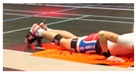	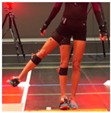	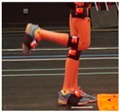	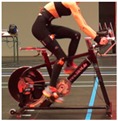
Unit vector identified for thigh	${}^{SU}Y_{BS}^{TH}$	${}^{LD}X_{BS}^{TH}$	${}^{HAA}X_{BS}^{TH}$		${}^{P}Z_{BS}^{TH}$
Unit vector identified for shank	${}^{SU}Y_{BS}^{SH}$	${}^{LD}X_{BS}^{SH}$		${}^{KFE}Z_{BS}^{SH}$	${}^{P}Z_{BS}^{SH}$

**Table 2:** Definition of the calibration methods—expressed as rotation matrices—by means of task combinations. For example, for the cycling method, the unit vector ${}^{SU-CY}X_{BS-IMU}^{TH}$ is obtained from the SU task and cycling task, and it defines the *X*-axis of the body segment frame for the thigh; the unit vector ${}^{SU-CY}Z_{BS-IMU}^{TH}$ defines the *Z*-axis of the body segment frame for the thigh; the unit vector ${}^{SU}Y_{BS-IMU}^{TH}$ is obtained from the standing up posture and it defines the *Y*-axis of the body segment frame for the thigh. Finally, the calibration matrix ${}^{C}R_{BS-IMU}^{TH/SH}$ is obtained by grouping the three unit vectors.

Method	First Task	Second Task	Thigh Frame	Shank Frame
$\mathrm{Reference}\;{}^{A}R_{BS-IMU}^{TH/SH}$	SU	P	XABS−IMUTHYABS−IMUTHZABS−IMUTH	XABS−IMUSHYABS−IMUSHZABS−IMUSH
$\mathrm{Static}\;{}^{S}R_{BS-IMU}^{TH/SH}$	SU	LD	$\begin{array}{c} {}^{SU-LD}X_{BS-IMU}^{TH}\\ {}^{SU}Y_{BS-IMU}^{TH}\\ {}^{SU-LD}Z_{BS-IMU}^{TH} \end{array}$	$\begin{array}{c} {}^{SU-LD}X_{BS-IMU}^{SH}\\ {}^{SU}Y_{BS-IMU}^{SH}\\ {}^{SU-LD}Z_{BS-IMU}^{SH} \end{array}$
$\mathrm{Mixed}\;{}^{M}R_{BS-IMU}^{TH/SH}$	SU	HAA/KFE	$\begin{array}{c} {}^{SU-HAA}X_{BS-IMU}^{TH}\\ {}^{SU}Y_{BS-IMU}^{TH}\\ {}^{SU-HAA}Z_{BS-IMU}^{TH} \end{array}$	$\begin{array}{c} {}^{SU-KFE}X_{BS-IMU}^{SH}\\ {}^{SU}Y_{BS-IMU}^{SH}\\ {}^{SU-KFE}Z_{BS-IMU}^{SH} \end{array}$
$\mathrm{Cycling}\;{}^{C}R_{BS-IMU}^{TH/SH}$	SU	P	$\begin{array}{c} {}^{SU-P}X_{BS-IMU}^{TH}\\ {}^{SU}Y_{BS-IMU}^{TH}\\ {}^{SU-P}Z_{BS-IMU}^{TH} \end{array}$	$\begin{array}{c} {}^{SU-P}X_{BS-IMU}^{SH}\\ {}^{SU}Y_{BS-IMU}^{SH}\\ {}^{SU-P}Z_{BS-IMU}^{SH} \end{array}$

**Table 3:** Mean differences in body segment orientations using the anatomical and technical frames. Differences were expressed as the smallest rotation angles for thigh and shank. Differences were also expressed as Euler angles around the anteroposterior *X*-axis, longitudinal *Y*-axis, and medio-lateral *Z*-axis for the thigh and shank. ** denotes a significant difference, with *p* < 0.01.

		Orientation Error for Each Method (Deg)			*p*-Values		
Segment	Angle	Static	Mixed	Cycling	S vs. M	S vs. C	M vs. C
Thigh	TOTAL	20.0 ± 6.6	22.2 ± 7.9	10.9 ± 1.6	0.78	0.003	0.001
	around X	−2.6 ± 2.2	−2.6 ± 2.2	−2.6 ± 2.2	n/a	n/a	n/a
	around Y	−16.2 ± 8.9	−19.1 ± 9.3	−1.7 ± 2.3	0.74	< 0.001	< 0.001
	around Z	−8.9 ± 2.1	−8.9 ± 2.1	−8.9 ± 2.1	n/a	n/a	n/a
Shank	TOTAL	17.4 ± 8.4	13.4 ± 3.5	11.8 ± 2.8	0.110	0.069	0.449
	around X	−4.6 ± 2.2	−4.6 ± 2.2	−4.6 ± 2.2	n/a	n/a	n/a
	around Y	−14.4 ± 9.8	−10.0 ± 4.3	−8.1 ± 2.5	0.152	0.548	0.089
	around Z	−5.9 ± 2.4	−5.9 ± 2.4	−5.9 ± 2.4	n/a	n/a	n/a

**Table 4:** Knee joint root mean squared (RMS) errors using each calibration method in comparison to the values obtained from the reference optoelectronic system and the ISB convention

	RMS Error in the Knee Angle for Each Method (deg)			*p*-Values		
DOF	Static	Mixed	Cycling	S vs. M	S vs. C	M vs. C
**Flexion/Extension**	4.79 ± 3.03	3.65 ± 2.23	3.74 ± 2.99	0.38	0.82	1
**Abduction/Adduction**	11.18 ± 6.62	7.51 ± 4.13	5.92 ± 2.85	0.062	0.035	0.346
**Internal/External Rotation**	15.37 ± 5.38	18.80 ± 8.05	6.65 ± 1.94	0.357	<0.001	<0.001
